# Serum Exosomal miR-941 as a promising Oncogenic Biomarker for Laryngeal Squamous Cell Carcinoma

**DOI:** 10.7150/jca.45394

**Published:** 2020-07-09

**Authors:** Qinli Zhao, Xiwang Zheng, Huina Guo, Xuting Xue, Yuliang Zhang, Min Niu, Jiajia Cui, Hongliang Liu, Hongjie Luo, Dongli Yang, Yong Shi, Hui Huangfu, Shuxin Wen, Yongyan Wu, Wei Gao, Binquan Wang

**Affiliations:** 1 Shanxi Key Laboratory of Otorhinolaryngology Head and Neck Cancer, Shanxi Medical University, Taiyuan 030001, Shanxi, P. R. China.; 2 Shanxi Province Clinical Medical Research Center for Precision Medicine of Head and Neck Cancer, The First Hospital of Shanxi Medical University, Taiyuan 030001, Shanxi, P. R. China.; 3 Department of Otolaryngology Head & Neck Surgery, The First Hospital of Shanxi Medical University, Taiyuan 030001, Shanxi, P. R. China.; 4 Department of Otolaryngology Head & Neck Surgery, General Hospital of Shenzhen University, Shenzhen 518061, Guangdong, P. R. China.; 5 Department of Biochemistry & Molecular Biology, Shanxi Medical University, Taiyuan 030001, Shanxi, P. R. China.; 6 Key Laboratory of Cellular Physiology, Ministry of Education, Shanxi Medical University, Taiyuan, 030001 Shanxi, P. R. China.

**Keywords:** laryngeal squamous cell carcinoma, exosome, biomarker, hsa-miR-941, diagnosis

## Abstract

At present, no blood-based biomarkers have been used in clinical practice for laryngeal squamous cell carcinoma (LSCC). Increasing evidence suggests that circulating exosomal microRNAs (miRNAs) may serve as potential diagnostic biomarkers for various cancers. This study aims to identify and evaluate serum exosomal miRNAs for LSCC diagnosis. The ExoQuick solution (EQ), which provides a high-yield and is a highly efficient exosome isolation method, was selected to isolate serum exosomes in the current study. In LSCC samples, exosome concentrations were higher than in healthy control (HC) samples. RNA-seq analysis identified a total of 1608 miRNAs, with 34 upregulated and 41 downregulated in LSCC samples relative to HC samples. Furthermore, qRT-PCR showed that miR-941 is significantly upregulated in LSCC serum exosomes, with this same trend seen in LSCC tissues and cells. Moreover, when examining miR-941 in cell lines, miR-941 overexpression promoted proliferation and invasion, while miR-941 knockdown inhibited cell proliferation and invasion. ROC curve analysis showed that miR-941 has an area under the curve (AUC) of 0.797 (95% CI = 0.676-0.918) for distinguishing LSCC patients from HCs. In conclusion, serum exosomal miR-941 may serve as a promising oncogenic biomarker for diagnosing LSCC, and has the potential as a therapeutic target.

## Introduction

Laryngeal squamous cell carcinoma (LSCC) is one of the most commonly seen malignant head and neck tumors, with a high incidence in northern China, including Shanxi Province 1, 2. While the incidences of LSCC have been declining in the past 40 years, the 5 year survival rate has been decreasing from 66% to 63% 3. LSCC patients usually have no overt clinical symptoms at the early stages, with around 60% of patients not diagnosed until an advanced stage (stage III or IV) 4. As is true with any malignant tumor, an increased chance of a successful treatment is highly dependent on an early diagnosis 5. In LSCC, early detection is very difficult given the anatomical location of the larynx and the tendency of a tumor to be more hidden. Furthermore, LSCC is predominantly diagnosed via endoscopy and pathological examination, which is a more invasive procedure and multiple biopsies are often required to reach a diagnosis. Therefore, it is urgent to develop a rapid, minimally invasive and highly sensitive diagnostic method for clinically diagnosing LSCC.

Exosomes are membranous vesicles with a diameter of 30-150 nm that contain proteins, lipids, and nucleic acids derived from their parental cells. They are released into various body fluids, including circulating blood, which is of clinical interest due to being minimally invasive and easily repeatable. The contents within exosomes have been found not to be randomly encapsulated, but reflect the state of the cells of origin 6, 7. Additionally, they can potentially reflect the structure and function of their parental cells, thus enabling the mapping of different pathophysiological states of the human body^8^ without the need to directly sample source cells 9, and subsequently can serve as potential tumor biomarkers 10, 11. Moreover, tumor cells have been shown to secrete more exosomes than normal cells, thus an increased exosome presence is seen in the blood of tumor patients relative to healthy people 12, 13. Collectively, these attributes make exosomes attractive tumor biomarkers.

MicroRNAs (miRNAs) are endogenous non-coding RNA molecules that are between 19-22 nucleotides long and are abnormally expressed in tumors, often acting as tumor suppressors or oncogenes 14, 15. MiRNAs have already been shown to be promising tumor biomarkers that are in many cases closely associated with tumor occurrence and development 16. In the case of circulating exosomal miRNAs, they are protected against nuclease digestion and are less susceptible to the influence of the extracellular environment; thus, they are able to provide more reliable insights 17. Moreover, since exosomal miRNA composition and abundance are determined by the state of the parental cell, these miRNAs can better reflect the functional status of the organ of origin. Additionally, some miRNAs are enriched in exosomes, which improves the detection of low abundance miRNAs 7, 8, 18. In several recent studies, circulating exosomal miRNAs have been shown to serve as early diagnostic and prognostic biomarkers, and aid in process monitoring in many tumors, including lung cancer, colorectal cancer, breast cancer, prostate cancer, and ovarian cancer 19-22. In LSCC, miR-21 and HOTAIR expression was quantified via quantitative reverse transcription PCR (qRT-PCR), and their combined detection was shown to serve as an important marker for screening laryngeal cancer patients 23. However, while this study did examine serum exosomal miRNAs, a more extensive examination of peripheral blood LSCC samples has not been performed.

When it comes to isolating exosomes from blood samples, there is currently no gold standard 24. Thus, this study first evaluated several current exosome isolation methods and found that ExoQuick solution (EQ) provides a higher yield and efficiency. Serum exosomes were then isolated from 6 LSCC patients and 6 healthy controls (HCs), and serum exosomal miRNA expression profiles were generated for each group using RNA sequencing (RNA-seq). Identified differential expression was subsequently validated via qRT-PCR and analyzed using receiver operating characteristic (ROC) curve analysis. The findings suggest that serum exosomal miR-941 may act as a potential LSCC diagnostic biomarker.

## Materials and Methods

### Patients and Clinical Samples

LSCC serum samples were collected at the Department of Otolaryngology Head and Neck Surgery of the First Hospital of Shanxi Medical University. HC subjects were screened by the Physical Examination Center of the hospital and for 12 months prior had no history of acute, chronic, or malignant disease or a surgical procedure. LSCC patients were histologically verified with no history of radiotherapy or chemotherapy and no evidence of an acute or chronic inflammatory disease. The HC samples were matched to LSCC patients by age and gender. There were 90 individuals enrolled in this study, including 59 LSCC patients and 31 HC subjects. For the exosome isolation optimization study, 3 LSCC patients were selected, while 6 LSCC and 6 HC subjects were included in the discovery set. Validation was performed using 50 LSCC patients and 25 HCs, of which 7 LSCC patients and 7 HCs were also included in the endogenous reference validation experiment. This study was approved by the Research Ethics Committee of Shanxi Medical University and each subject provided informed consent.

Peripheral blood samples were collected in serum separator tubes and processed within 2 hours after collection. The blood samples were centrifuged at 1,200 g for 10 minutes at 4 °C and the obtained supernatant was aspirated and centrifuged at 3,000 g for 15 minutes at 4 °C. Finally, the supernatant was broken into multiple aliquots and stored at -80 °C.

### Serum Exosome Isolation

#### Exosome Isolation by Ultracentrifugation (UC)

Exosomes were isolated using the UC method as previously described 25. Briefly, serum samples (n = 3) were thawed on ice and 1 mL of serum was diluted in 11 mL PBS. Samples were then ultracentrifuged at 150,000 g overnight at 4°C. Next, the supernatant was discarded, the precipitate was washed in 11 mL PBS, and the samples underwent another ultracentrifugation at 150,000 g for 2 hours at 4°C. Finally, the supernatant was discarded, and the precipitate was resuspended in PBS.

#### Exosome Isolation by ExoQuick (EQ) Solution

ExoQuick exosome precipitation solution (System Biosciences, Mountain View, CA, USA) was used as per the manufacturer's instructions. Briefly, serum was combined with EQ solution (4:1) and incubated at 4 °C for 30 minutes. The ExoQuick/serum mixture was then centrifuged at 1,500 g for 30 minutes at 4 °C and then centrifuged for 5 minutes, with the supernatant removed each time. The obtained pellets were resuspended in PBS.

#### One Step Exosome Isolation Using 8% Polyethylene Glycol (PEG1)

Exosomes were isolated using PEG as previously described at a final concentration of 8% due to this concentration being shown to provide the highest yield without sacrificing purity 26. The PEG solution was prepared by combining 8 g PEG (6 kDa, Sigma), 2.922 g sodium chloride (NaCl) and 50 mL deionized water, which was then filtered through a 0.22 μm-pore filter to make a 16% PEG (g/mL) solution. The 16% PEG solution was then combined with serum (1:1) and mixed by inversion to achieve a final concentration of 8% and incubated at 4 °C overnight. The samples were then centrifuged at 3,500 g for 1 hour at 4 °C, the supernatant was discarded, and the pellet was resuspended in PBS.

#### Two Step Exosome Isolation Using 8% PEG + 5% PEG (PEG2)

In the study of Rider et al.^26^, in order to further remove serum proteins that co-precipitated with vesicles and purify the obtained vesicles, the exosome samples resulting from the PEG1 method were re-pelleted by PEG-precipitation for a second time using a lower concentration of PEG (5%), or by PBS wash by ultracentrifugation (100,000 g). We chose a secondary PEG treatment here, because this method is easy to operate in any laboratory, but the ultracentrifuge is not indispensable equipment in the laboratory. For this isolation method, the PEG1 method above, which requires a 16% PEG solution, is combined with a second step that requires a 10% PEG solution. The 10% PEG solution was prepared as described above, but with 5 g of PEG used. After completing the 8% PEG step, the obtained pellet was resuspended in 100 μL PBS and further diluted in 5 mL PBS. The obtained sample was then combined with 10% PEG solution (1:1) to give a final PEG concentration of 5%. This sample was then incubated at 4 °C overnight and centrifuged at 3,500 g for 1 hour. The supernatant was discarded, and the pellet was resuspended in PBS.

### Characterization and Quantification of Exosomes

#### Transmission Electron Microscope (TEM)

The exosome samples were diluted with an appropriate volume of PBS. Diluted exosome samples (10 μL) were applied dropwise onto copper grids for 1 minute, followed by staining with 2% uranyl acetate (10 μL) applied dropwise for 1 minute. Any excess was removed by blotting with filter paper following each step. The grids were air dried for 15 minutes and imaged at a voltage of 120 kV on a FEI Tecnai G2 spirit TEM (Thermo-Fischer, Waltham, MA, USA).

#### Exosome Marker Detection via Western blot and Antibody Array

Exosome samples were lysed in RIPA buffer (Thermo-Fischer, Waltham, MA, USA) with protease inhibitor (Thermo-Fischer), and protein concentrations were measured using a BCA protein assay kit (Yeasen Biotechnology, Shanghai, China). Protein samples (70 μg) were resolved via 8-16% polyacrylamide electrophoresis and transferred to a PVDF membrane (Millipore, Boston, MA, USA). The PVDF membranes were then blocked for 1 hour at room temperature with 5% non-fat milk power and incubated overnight at 4 °C with one of the following primary antibodies: anti-CD63 (1:500; #ab59479, Abcam, Cambridge, MA, USA), anti-CD81 (1:100; #sc-166029, Santa Cruz, Dallas, TX, USA), and anti-TSG101 (1:100; #sc-7964, Santa Cruz). The next day, membranes were in PBS and incubated with horseradish peroxidase-labeled secondary antibody (1:1000, #A0216, Beyotime, Shanghai, China) for 2 hours at room temperature. The blots were then rinsed with PBS, visualized with a chemiluminescent reagent (Advansta, Menlo Park, CA, USA), and imaged.

An Exo-Check Exosome Antibody Array (System Biosciences, Mountain View, CA, USA) was also used to detect the presence of several exosomal markers as per the manufacturer's instructions. Briefly, 300 µg protein was incubated with 600 µL Exosome Lysis buffer and vortexed. Next, the lysate mixture was combined with 9.4 mL Exosome Array Binding buffer, added to a pre-wet antibody array membrane, and incubated overnight at 4 °C on a shaker. The next day, the membrane was washed, and 10 mL Detection buffer was added and allowed to incubate for 2 hours on a shaker. Finally, the membrane was washed and imaged.

#### Nanoparticle Tracking Analysis (NTA)

To determine exosome size and concentration distributions, NTA was performed using a Nanosight NS300 system with NTA 2.3 Software (Malvern Instruments Ltd, Malvern, UK) as per the manufacturer's instructions.

#### Serum Exosomal RNA Extraction and Sequencing Library Preparation

Serum exosomal RNA was extracted using TRIzol reagent, with 10 ng glycogen added to facilitate precipitation and then isopropanol was added and allowed to precipitate overnight at -20˚C. The RNA purity, concentration and integrity were determined using a NanoPhotometer (Implen, München, Germany), Qubit 2.0 (Life Technologies, Thermo Fisher Scientific Inc.) and Agilent 2100 Bioanalyzer (Agilent Technologies, Santa Clara, CA, USA), respectively. Small RNA libraries were generated using a NEBNext® Multiplex Small RNA Library Prep Set for Illumina® (NEB, Ipswich, MA, USA) as per the manufacturer's recommendations, with index codes added to attribute sequences to a given sample. PCR products were purified on an 8% polyacrylamide gel (100V, 80 minutes), with DNA fragments between 140 and 160 bp (the length of small noncoding RNA plus the 3' and 5' adaptors) excised and dissolved in 8 μL of elution buffer. The quality of each library was assessed using DNA High Sensitivity Chips (Agilent) on an Agilent Bioanalyzer 2100 system. The library preparations were sequenced on an Illumina Hiseq 2500 platform and 50 nt single-end reads were generated.

### Bioinformatics Analysis of RNA-seq Data

To ensure high sequence quality, raw data was filtered by removing reads containing ploy-N, 5' adapter contaminants, those lacking a 3' adapter or insert tag, reads containing ploy-A, -T, -G or -C, or low-quality reads to leave only clean reads. Clean reads with a length of 18-35 nt were selected as small RNA for subsequent analysis. Small RNAs (sRNAs) were mapped to human reference gene hg19 using Bowtie. Matched sRNAs were then blasted against miRbase20.0 to identify known miRNAs and analyzed using miRDeep2 and miREvo to predict novel miRNAs. The miRNA expression levels were then calculated and normalized to TPM (transcript per million). Differential expression analysis was performed by using the DESeq R package (1.8.3), with a significance threshold of P < 0.05 and | log2 fold-change | ≥ 0.5. For the identified differentially expressed miRNAs, target genes prediction was performed using miRanda, PITA and RNAhybrid, and KOBAS was used to examine their statistical enrichment within KEGG pathways.

### Identification of Endogenous References

Candidate reference genes were selected based on our RNA-seq data and the literature. MiRNAs with stable expression based on RNA-seq data, having a small coefficient of variation (CV) value and having a moderate expression level were evaluated as candidate reference miRNAs. Candidate reference expression stability was then statistically analyzed using several statistical algorithms, including BestKeeper 27, NormFinder 28, geNorm 29, ∆Ct method 30, and RefFinder 31.

### miRNA Quantification by qRT-PCR

For cDNA synthesis, an All-in-One™ miRNA First-Strand cDNA Synthesis Kit (Genecopoeia, Rockville, MD, USA) was used per the manufacturer's protocol. Reverse transcription reactions were completed by incubating the mixtures at 37°C for 60 minutes, at 85°C for 5 minutes, and then storing it at 4°C. The qRT-PCR was performed using ChamQ SYBR qPCR Master Mix (Vazyme, Nanjing, China) according to the manufacturer's protocol, with all reactions examined in triplicate. The forward primers of miRNAs were designed by using miRprimer2 software 32 and synthesized by Nanjing GenScript Co. The universal reverse primers were provided by the All-in-One kit. All primer sequences are listed in Table 1. The qRT-PCR reactions were completed on a StepOnePlus Real-Time PCR System (Applied Biosystems, Waltham, MA, USA) under the following conditions: 95°C for 3 minutes, and then 40 cycles at 95°C for 10 seconds and 60°C for 30 seconds. Each qRT-PCR value was normalized to the geometric mean of the endogenous references (miR-30a-5p, miR-532-5p and U6) and relative expression was determined by using the 2^-ΔCt^ method. All raw data was log10 transformed.

### Cell Culture, Transfection and Cell-derived Exosome Isolation

The human LSCC cell line FD-LSC-1 was a gift from Professor Liang Zhou and was cultured in BEGM™ Bronchial Epithelial Cell Growth Medium (Lonza, Walkersville, MD, USA) supplemented with 10% FBS (BI, Cromwell, CT, USA) 33. A LSCC cell line Tu 686 and normal human oral keratinocytes cell line (HOK), were supplied by Bioleaf Biotech Company (Shanghai, China). These two cell lines were maintained in DMEM (Gibco, Grand Island, NY, USA) supplemented with 10% FBS (BI, Cromwell, CT, USA) and 1% penicillin/streptomycin (Solarbio, Beijing, China) at 37°C and 5% CO_2_. MiR-941 mimics, miR-941 inhibitor, and corresponding negative controls (NC) were designed and synthesized by GenePharma (Shanghai, China). Target cells were transfected with miR-941 mimics (50 nM), miR-941 inhibitor (150 nM), or the corresponding NC using Lipofectamine 3000 (Invitrogen, CA, USA) according to the manufacturer's protocol. To isolate cell-derived exosomes, cells were cultured in DMEM or BEGM with 10% exosome-depleted FBS (VivaCell, China) for 48 hours. Exosomes were then isolated using ExoQuick-TC exosome precipitation solution (System Biosciences, Mountain View, CA, USA) according to the manufacturer's protocol.

### Cell Proliferation Assay

Cell proliferation was measured by using a cell-counting kit-8 (CCK-8) assay (TransGen Biotech, Beijing, China) according to the manufacturer's instructions. Transfected cells were examined at 24, 48, and 72 hours post-transfection, with 10 μL of CCK8 added to the medium and allowed to incubate for 1 hour. Absorbances were then measured at a wavelength of 450 nm.

### Cell Invasion Assay

A cell invasion assay was performed by using 24-well Transwell chambers with 8 µm pores (BD Biosciences, San Jose, CA, USA). Matrigel (50 μL; BD Biosciences) diluted 1:6 in serum-free medium was added to the upper chamber of the Transwell insert and incubated for 2 hours at 37 °C. Transfected cells (1 × 10^5^ cells/well) were seeded into the top chamber in serum-free medium, with medium containing 10% FBS added to the bottom chamber. After 24 hours, the upper side of the filter membrane was wiped with a cotton swab to remove any non-invading cells. The cells on the lower side of the filter were fixed with 4% paraformaldehyde for 20 minutes and stained with crystal violet for 10 minutes. A cell count was obtained by counting five random microscopic fields (100× magnification) per filter.

### Statistical Analysis

Statistical analysis was performed by using the SPSS statistical package (version 22; SPSS, Chicago, IL, USA). The data were displayed as a mean ± SD for samples or duplicate wells where applicable. Statistical significance between two groups was assessed by using a Student's *t*-test (for independent samples) or Mann-Whitney test (non-parametric), and a Kruskal-Wallis test was used when comparing three groups. Serum exosomal miRNA levels were expressed as log10 (2^-ΔCt^) and receiver operating characteristic (ROC) curves were generated based on the ΔCt values. All tests were two-tailed and results were considered statistically significant at *P* < 0.05.

## Results

### EQ method provides high-yields and highly efficient exosome isolation

**Figure 1:**
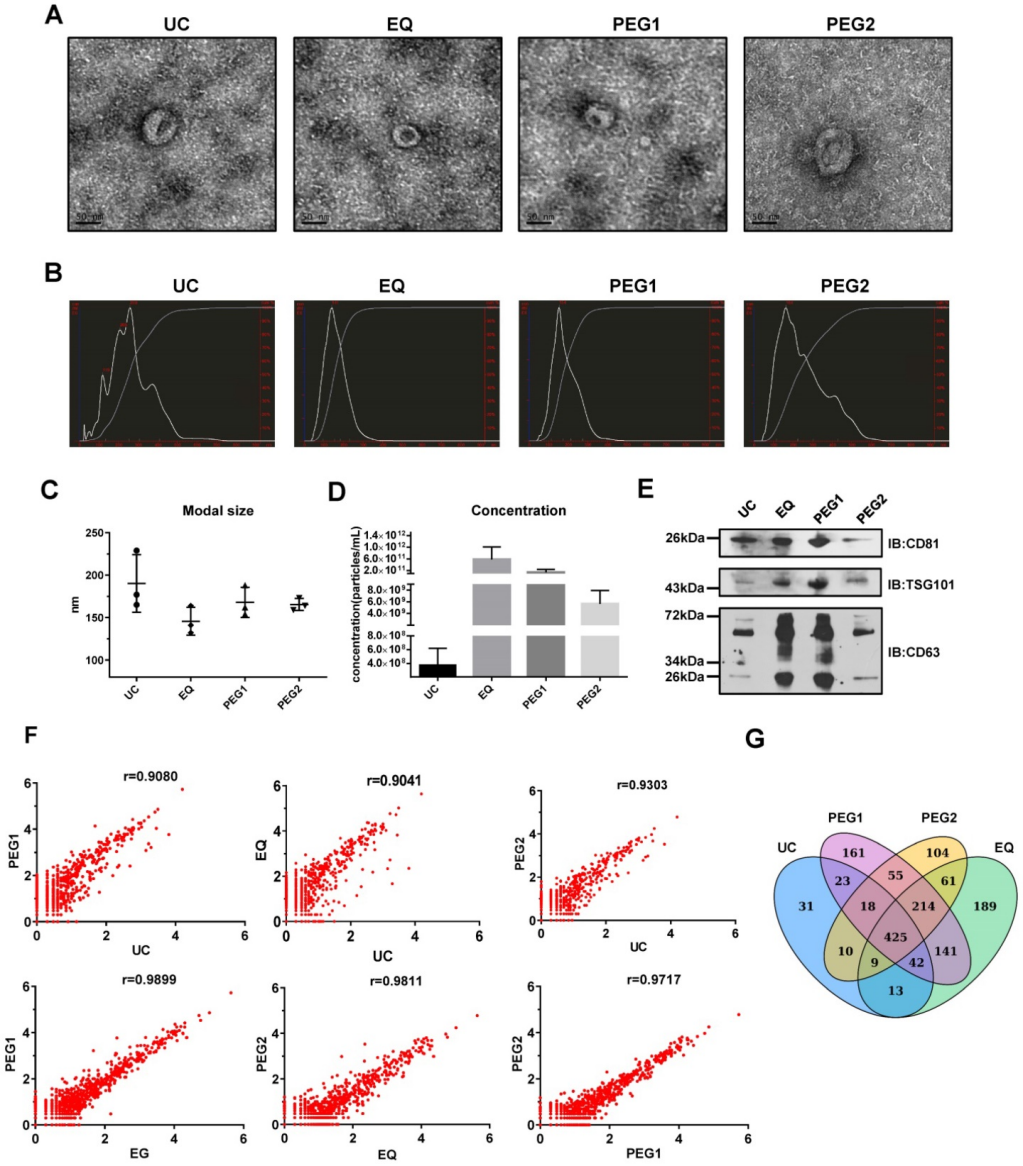
** Characterization and miRNA Profiles for Three LSCC Serum Exosomal Samples Isolated with Four Different Isolation Methods.** (**A**) Representative transmission electron microscope (TEM) images for each extraction method. Scale bars = 50 nm. (**B**) Representative size distribution profiles obtained with Nanoparticle Tracking Analysis (NTA). (**C**) Modal sizes (nm) and (**D**) concentrations (particles/mL) of exosome samples examined via NTA. (**E**) Western blot analysis of exosomal markers (CD81, TSG101 and CD63) in lysates obtained by using the four different isolation methods. (**F**) Scatterplots of miRNAs RNA-seq expression profiles. Pearson correlation coefficient (r) was used as a measure of the strength of the linear relationship between the two exosomes samples obtained with two different methods. (**G**) Venn diagram showing unique and shared miRNAs between the UC, EQ, PEG1 and PEG2 samples.

To identify an exosome isolation method that is optimal regarding ease and reproducibility, blood samples from 3 LSCC patients were utilized and four isolation methods, including UC, EQ, PEG1 and PEG2, were evaluated by examining morphology, size, concentration, protein markers and miRNA profiles. The starting volume of serum, which was used to extract exosomes by four methods, was equal. TEM measurements showed that all the methods enriched exosomes with a typical cup-shaped morphology (Figure 1A). To assess exosome size and concentration, NTA was performed. The results showed that for the EQ method, the vesicles harvested at the peak level generally had a modal size of less than 150 nm (145.7 ± 16.5 nm), while the others were above 150 nm at 165.3 ± 7.1 nm (PEG2), 190.3 ± 34.0 nm (UC), and 168.0 ± 17.8 nm (PEG1). Furthermore, the UC and PEG2 methods displayed multiple particle size peaks, with some larger particles > 300 nm also noted (Figure 1B and 1C). Additionally, the EQ method was shown to provide the highest exosome concentration of the methods (5.82E+11 particles/mL; Figure 1D), followed by PEG2 (5.64E+09 particles/mL), UC (3.73E+08 particles/mL), and PEG1 (1.34E+11 particles/mL).

Next, exosomal markers, CD63, CD81 and TSG101, were examined via Western blot and were found to be enriched for all 4 methods (Figure 1E). However, while the same amount of total protein was loaded into the gel, expression levels for these markers were higher in samples extracted with the EQ and PEG1 methods relative to the UC and PEG2 methods. Prior to performing RNA-seq to examine miRNA profiles associated with each extraction method, the three LSCC exosomal RNA samples were pooled. Similarities between the four miRNA profiles were examined and a Pearson correlation coefficient above 0.90 was obtained (Figure 1F), with a total of 571 (UC), 1094 (EQ), 1079 (PEG1), and 896 (PEG2) miRNAs detected (Figure 1G). Overall, the EQ method showed a higher yield and efficiency, with comparatively rapid processing; thus, it was selected to isolate exosomes in subsequent experiments.

### LSCC patients have higher serum exosome levels than HC subjects

**Figure 2:**
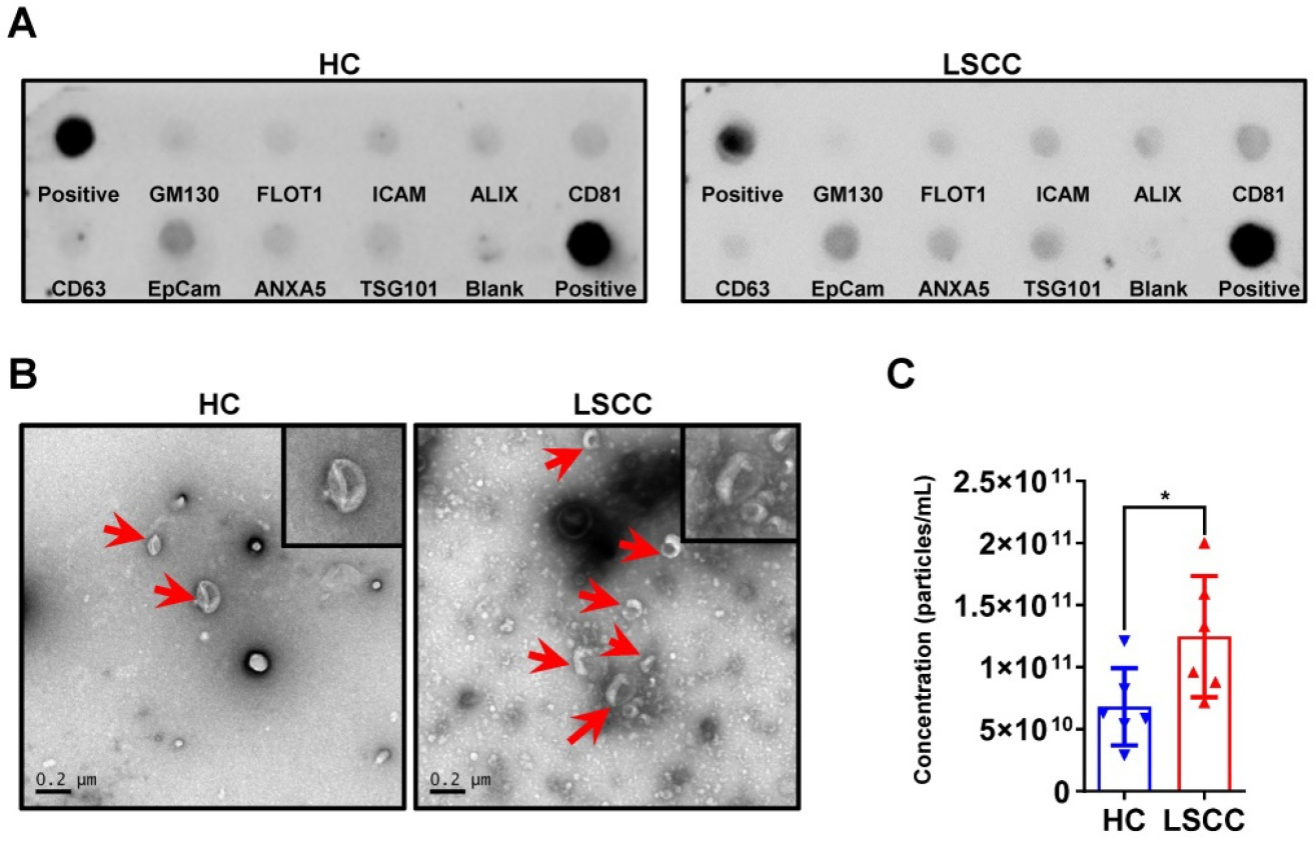
** LSCC Serum Exosome Levels are Higher Relative to the HC Samples.** (**A**) Representative Exo-Check Exosome Antibody Array for detecting exosomal markers (CD81, CD63, ALIX, FLOT1, ICAM, EpCam, ANXA5 and TSG101) and assessing cellular contamination (cis-Golgi matrix-associated protein GM130). (**B**) Representative TEM images of serum exosomes in LSCC and HC samples. Red arrows indicate exosomes, scale bars = 0.2 µm. (**C**) Serum exosome concentration (particles/mL) in LSCC and HC samples as detected with NTA. Data are presented as a mean ± standard deviation (SD). *P < 0.05.

Serum exosomes were extracted from 6 LSCC patients and 6 HCs using the EQ method; sample quality and exosome levels were examined. First, exosomal markers were examined using an Exo-check antibody array and showed that for both groups CD81, CD63, ALIX, FLOT1, ICAM, EpCam, ANXA5 and TSG101 were expressed, but GM130 (cis-Golgi matrix-associated Protein), a cellular contamination marker, was not expressed (Figure 2A). Next, serum exosome levels were examined using TEM and NTA. The TEM analysis showed that LSCC samples have higher exosome levels than HC samples when comparing equal serum volumes (Figure 2B). This finding was further confirmed by NTA, which showed that the relative exosome concentration in LSCC serum is significantly higher than the levels in HCs (Figure 2C).

### LSCC patients have different miRNA profiles from HCs

**Figure 3:**
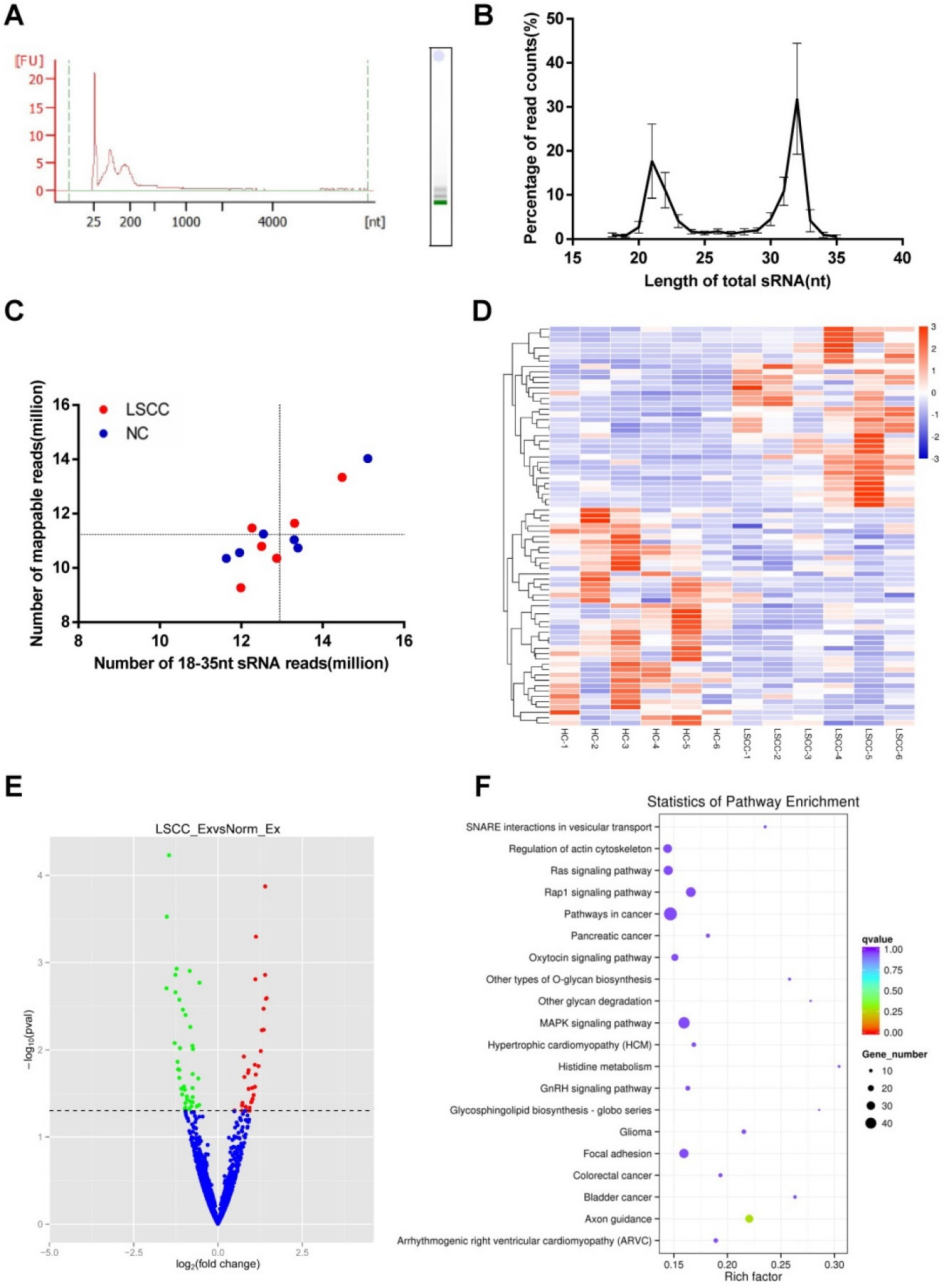
** RNA-seq Analysis of the Discovery Set Including 6 LSCC and 6 HC Samples.** (**A**) Analysis of serum exosomal RNA using an Agilent 2100 and electrophoresis indicated a significant population of small RNAs and an absence of 18S and 28S RNAs. (FU, fluorescence units; nt, nucleotides). (**B**) Length distribution of sequenced small RNAs (sRNAs). (**C**) Number of 18-35 nt sRNA reads vs. number of mapped reads. The horizontal and vertical lines are the mean levels of mapped reads and sRNA reads, respectively. (**D**) Hierarchical clustering of the differentially expressed LSCC and HC miRNAs. (**E**) Volcano plot identifying 34 significantly upregulated (red dots) and 41 downregulated (green dots) miRNAs. The horizontal line represents a *P-*value of 0.05, with the differentially expressed cut-off threshold set to *P* < 0.05 and | log2 fold-change | ≥ 0.5. (**F**) KEGG pathway analysis scatter plot of the predicted target genes associated with the differentially expressed miRNAs.

To further compare LSCC (n = 6) and HC (n = 6) samples, serum exosomal miRNA profiles were constructed using RNA-seq. Across samples, the results showed that the RNAs are predominantly small RNA species, with no detectable 18S or 28S ribosomal RNA (Figure 3A). On average, RNA-seq generated 13.8 million raw reads per sample, with a range between 12.4 to 15.9 million. Of these, 95-98% of the reads were high-quality clean reads, and only sRNAs with a length of 18-35 nt were selected for subsequent analysis. Read counts and size distributions were then examined, with two predominant peaks at 21 nt and 32 nt identified (Figure 3B). As exosomes contained degradation fragments of other RNAs, the reads counts were higher at 30-32 nt. The identified sRNA reads were then examined using Bowtie, with 77-94% of the reads mapped to reference RNA sequences (Figure 3C).

The 1,608 miRNAs were identified and then examined using DESeq to identify differential expression between the LSCC and HC miRNAs. Hierarchical cluster analysis showed that LSCC miRNA expression patterns are significantly different from the HC samples (Figure 3D). A volcano plot was also constructed to identify significantly differentially expressed miRNAs (*P* < 0.05, | log2 fold-change | ≥ 0.5). The results identified 34 upregulated and 41 downregulated miRNAs in LSCC individuals relative to the HCs (Figure 3E, Table 2).

To predict target genes for the differentially expressed miRNAs, miRanda, PITA and RNAhybrid were utilized. To examine the statistical enrichment of these identified target genes within KEGG pathways, KOBAS was utilized. Pathway analysis revealed that the predicted target genes are mainly involved in cancer/tumor related pathways, including MAPK, Ras, and focal adhesion signaling pathways (Figure 3F).

### miR-30a-5p, miR-532-5p and U6 as best endogenous reference genes

**Figure 4:**
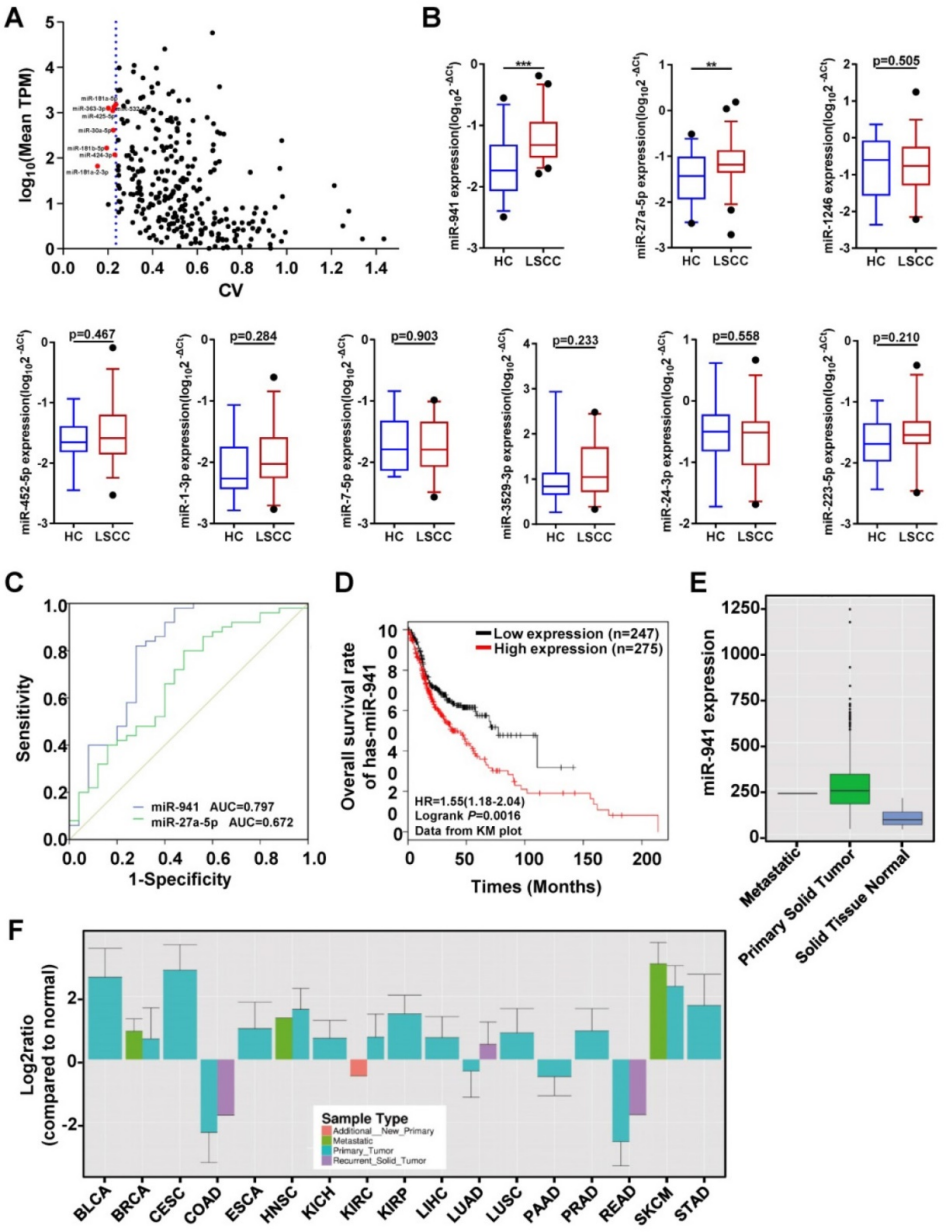
** Quantitative Analyses of miRNAs in Clinical Samples and Receiver Operating Characteristic (ROC) Curve Analysis.** (**A**) Candidate endogenous references selected from the discovery set (n = 12) RNA-seq data. The scatter plot shows expression level distributions normalized to TPM (transcript per million) and the coefficient of variation (CV) values. The y-axis represents the mean miRNA expression levels and is displayed as log10 (mean TPM). The x-axis represents the dispersion degree of these miRNAs and is described by the CV. The most stably expressed miRNAs with a moderate expression are indicated with red dots (n = 8). The blue dotted line represents the maximum CV value of these 8 miRNAs (0.236). (**B**) A subset of 9 differentially upregulated miRNAs identified from the RNA-seq data were further examined using an independent validation set (50 LSCC patients and 25 HCs), with expression levels visualized using box plots. MiRNA expression levels were detected using qRT-PCR, with selected endogenous references (miR-30a-5p, miR-532-5p and U6) used as controls. The Y-axis displays the expression level as log10(2^-ΔCt^). *** P < 0.001, ** *P* < 0.01. (**C**) ROC curves assessing LSCC miR-941(blue line) and miR-27a-5p (green line), with area under the curve (AUC) values also determined. (**D**) Kaplan-Meier survival curves examining miR-941 expression in head and neck squamous cell carcinoma (HNSCC) tissue sample data obtained from the TCGA database. MiR-941 was significantly correlated with a poor outcome (log rank test, *P* = 0.0016). (**E**) Examination of HNSCC primary solid tumor data obtained from the YM500v3 database showed increased miR-941 expression. (**F**) Expression levels of miR-941 in different tumors from the YM500v3 database. The Y-axis represents the relative expression level in the tumors as compared to normal tissues. The X-axis displays the different tumor types. BLCA, Bladder urothelial carcinoma; BRCA, Breast invasive carcinoma; CESC, Cervical squamous cell carcinoma and endocervical adenocarcinoma; COAD, Colon adenocarcinoma; ESCA, Esophageal carcinoma; HNSC, Head and Neck squamous cell carcinoma; KICH, Kidney chromophobe; KIRC, Kidney renal clear cell carcinoma; KIRP, Kidney renal papillary cell carcinoma; LIHC, Liver hepatocellular carcinoma; LUAD, Lung adenocarcinoma; LUSC, Lung squamous cell carcinoma; PAAD, Pancreatic adenocarcinoma; PRAD, Prostate adenocarcinoma; READ, Rectum adenocarcinoma; SKCM, Skin cutaneous melanoma; STAD, Stomach adenocarcinoma.

To identify serum exosomal miRNAs from the RNA-seq data that are consistently expressed in both LSCC and HC samples, the method reported by Zhan et al.^34^ was utilized with some adjustments. First, miRNAs with significantly different expression levels between the two groups (*P* < 0.05), or if a mean TPM < 1 was obtained, were excluded. Second, mean TPMs were compared between the two groups and miRNAs with a ratio < 0.75 or > 1.3 were excluded. Finally, CVs were calculated and ranked from smallest to largest, and the top eight miRNAs with a TPM > 50 were selected as candidate internal reference genes (Figure 4A). Thus, eight candidate reference miRNAs, including miR-30a-5p, -532-5p, -181a-5p, -425-5p, -363-3p, -424-3p, -181b-5p, and -181a-2-3p, and two previously published endogenous controls, U6 and miR-16-5p, were selected 18, 21, 23, 35-38.

These candidate references were then further evaluated using qRT-PCR, with another 7 LSCC and 7 HC serum exosome samples utilized. Among them, miR-181a-2-3p was excluded due to its low expression and having a Ct value > 33. The remaining 9 candidate references were then examined using five different statistical algorithms to examine the expressional stability. While some of the stability rankings between the different algorithms varied, all five algorithms ranked the first four genes as miR-30a-5p, -532-5p, -181a-5p and U6 (Table 3). Previous studies have suggested the use of three reference genes 29, 39, and as such, miR-181a-5p was removed due to having the lowest expression level of the four and the highest Ct value = 31.

### Upregulated miRNAs further validated in a larger independent set

The main aim of this study was to identify LSCC diagnostic biomarkers, with upregulated miRNAs focused on since they are more easily applied clinically. Of the 34 differentially upregulated miRNAs, 9 with a TPM value ≥ 50 were selected for further validation in another independent set that included 50 LSCC and 25 HC individuals (Figure 4B). For 7 of the miRNAs, namely miR-1246, -452-5p, -1-3p, -7-5p, -3529-3p, -24-3p, and -223-5p, no statistical differences were observed. However, miR-941 and miR-27a-5p expression levels were significantly higher in LSCC patients relative to the HCs.

To further examine the discriminative ability of miR-941 and miR-27a-5p, ROC curve analysis was performed. The results showed that the area under curve (AUC) value for miR-941 was 0.797, with a 95% confidence interval (CI) = 0.676-0.918, while miR-27a-5p had an AUC = 0.672 and 95% CI = 0.54-0.804 (Figure 4C; Table 4). When comparing the AUC values, the higher cutoff for miR-941 (> 0.7) indicated that it would be a more ideal LSCC marker, thus it was further examined.

Next, potential correlations between serum exosomal miR-941 expression and LSCC clinicopathological characteristics were examined in the above 50 LSCC patients. No significant association between miR-941 expression and patient age, sex, T staging, cervical lymph node metastasis, clinical stage or pathologic differentiation were noted (Table 5). To further examine the expression level of miR-941 in LSCC tissues, miRNAs profiles for 57 pairs of LSCC tissues and corresponding normal tissues that we previously sequenced were examined and miR-941 was found to be 2.1-fold upregulated in LSCC tissues (*P* < 0.001) (Table 6)**.** Next, miR-941 expression was screened against the Ym500v3 40 database, and miR-941 expression was also found to be significantly increased in the tumor tissues of head and neck squamous cell carcinoma (HNSCC) relative to normal tissues (Figure 4E). Furthermore, data from Kaplan-Meier plotter database showed that upregulated miR-941 is significantly correlated a poor outcome for HNSCC patients (Figure 4D). Because the follow-up data of LSCC patients in this study were incomplete, survival analysis could not be carried out. Similarly, when examining miR-941 expression overall within the Ym500v3 database, it was shown to be significantly highly expressed in most tumor tissues relative to normal tissues, including lung squamous cell carcinoma, esophageal cancer, cervical squamous cell carcinoma, skin cutaneous melanoma, liver hepatic carcinoma, and breast carcinoma (Figure 4F).

**Figure 5:**
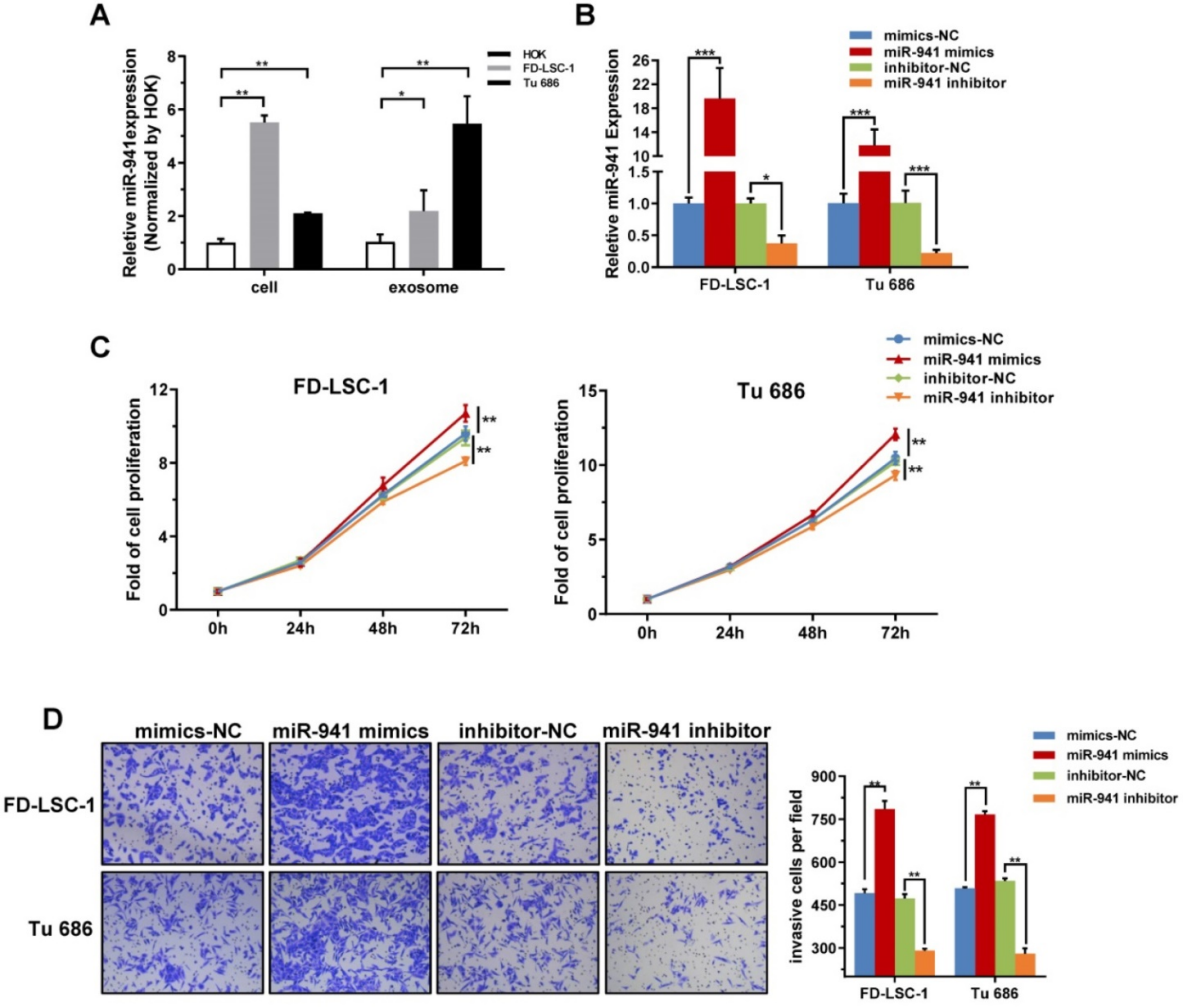
** miR-941 is Highly Expressed in LSCC Cell Lines and Promotes Proliferation and Invasion.** (**A**) Cellular and exosomal RNA was extracted from two LSCC cell lines (FD-LSC-1 and Tu 686) and a normal cell line (HOK), and miR-941 levels were quantified via qRT-PCR. Data were normalized to levels of U6 and compared with the nontumor cell line HOK. (**B**) MiR-941 expression was detected in FD-LSC-1 and Tu 686 by qRT-PCR after transfection of miR-941 mimics, miR-941 inhibitor, or associated controls. (**C**) A CCK8 assay was performed to determine the effect of miR-941 gain or loss on cell proliferation. (**D**) The effect of miR-941 gain or loss on cell invasion was evaluated by using a Transwell invasion assay. Representative images are shown. Cell numbers were counted by randomly selecting five fields at 100× magnification. Data are presented as a mean ± SD. ***P < 0.001, **P < 0.01, *P < 0.05.

Previous studies have reported that miR-941 is preferentially expressed in proliferating cells, tumor cells and tumor tissues, and targets many tumor suppressor genes 41, 42. To evaluate the expression of miR-941 in LSCC cells, miR-941 levels were detected in two LSCC cell lines, FD-LSC-1 and Tu 686, and a normal human oral keratinocyte line, HOK, by qRT-PCR. The result showed that miR-941 was significantly upregulated in both LSCC cell lines (Figure 5A). Additionally, exosomal RNA was extracted from the conditioned media for each cell line and exosomal miR-941 expression in FD-LSC-1 and Tu 686 cells was elevated relative to the HOK cells. When combining our experimental results and publicly available data, we speculate that miR-941 may regulate the malignant behavior of LSCC cells.

### miR-941 overexpression promotes LSCC cell proliferation and invasion

Malignant proliferation and local invasion are the main malignant behaviors of LSCCs, thus miR-941 potentially functioning in these areas was examined. FD-LSC-1 and Tu 686 cells were transfected with miR-941 mimics or inhibitor, with the transfection efficiency verified using qRT-PCR (Figure 5B). CCK-8 assays showed that overexpressing miR-941 significantly increases cell proliferation, while those transfected with miR-941 inhibitor have a repressed proliferation (Figure 5C).

Furthermore, the Transwell invasion assay showed that miR-941 overexpression enhances the invasion ability of FD-LSC-1 and Tu 686 cells, while miR-941 knockdown inhibits invasion (Figure 5D). Overall, these findings indicate that miR-941 can act as an oncogene to enhance LSCC cell proliferation and invasion.

## Discussion

This study began by comparing four exosomal isolation methods, with the EQ method selected as being the most optimal. Next, serum exosomes were successfully isolated from LSCC and HC samples, and LSCC serum exosome levels were found to be elevated relative to the HCs. Furthermore, RNA-seq data showed that the LSCC serum exosomal miRNA profiles are different from those of HC samples. After analyzing these findings, miR-941 was isolated and shown to be a potentially useful LSCC diagnostic marker following qRT-PCR validation and ROC curve analysis. Finally, cell functional experiments suggested that miR-941 may play an oncogenic role by promoting LSCC cell proliferation and invasion.

At present, there are several methods for extracting exosomes based on different principles, with each having its own advantages and drawbacks 43. Currently, there is no standardized high-yield and highly efficient exosome isolation method, especially when isolating from viscous blood samples. Herein, four isolation methods were compared, with exosome characteristics, including shape and size, concentration, markers and miRNA profiles, examined. While all of the obtained miRNA profiles were strongly correlated (r > 0.90) regardless of method, some of the methods were less desirable for other reasons. For example, the UC and PEG2 methods were time consuming, provided low yields, and produced vesicles that were slightly larger. For the PEG1 method, vesicle size and concentration were acceptable, but the overnight extraction was time consuming. In the future, we will try to shorten the PEG1 isolation time as suggested in another study to see if improved results are obtained 44. After all, for an exosomal isolation method to be applied clinically, the research cost will also have to be reduced. After a comprehensive analysis, the EG method was selected as it was shown to optimally isolate serum exosomes and can effectively process a large number of clinical samples in a short period of time with only a small amount of sample volume 45. Using the EQ method, serum exosomes were isolated from LSCC and HC samples, and TEM and NTA analyses showed that exosome concentrations are significantly higher in LSCC patients relative to HCs, indicating that exosomes could be potential tumor biomarkers.

In view of their high stability and source cell specificity, circulating exosomal miRNAs have been eagerly sought as biomarkers for cancer diagnosis and prognosis. In non-small cell lung cancer (NSCLC), plasma exosomal miRNAs were examined using RNA-seq and qRT-PCR, and the results showed that these miRNAs can be used to differentiate NSCLC patients from heathy individuals. Additionally, adenocarcinoma-specific and squamous cell carcinoma-specific exosomal miRNAs have been identified as potential biomarkers 20. In a study examining castration-resistant prostate cancer (CRPC), circulating exosomal miRNAs were examined using RNA-seq and qRT-PCR and found to be associated with overall survival, with miR-1290 and miR-375 identified as promising CRPC prognostic biomarkers 46. In laryngeal cancer, no previous studies have obtained a circulating exosomal miRNA profile. In this study, LSCC serum exosomal miRNA profiles were generated using RNA- seq and 75 differentially expressed miRNAs were identified, with their target genes found to be mainly associated with cancer related pathways. While RNA-seq can quantify all of the miRNAs present in a sample with a high level of accuracy and sensitivity, it is not a suitable platform for large scale screening 47. Herein, RNA-seq was only used to isolate potential markers using a small discovery set, and the findings were then adapted for qRT-PCR. To facilitate clinical application and improve the detection rate, the 34 differentially upregulated miRNAs were narrowed to only 9 that were further evaluated. Following additional qRT-PCR and ROC curve analysis, 7 of the miRNAs showed no differential expression, which left miR-941 and miR-27a-5p. MiR-27a-5p was found to have a low diagnostic value and a low AUC value (0.672), while miR-941 had a higher AUC value (0.797); thus indicating that miR-941 can provide a moderate level of accuracy with a reasonable capacity to identify different populations 48, 49. Subsequently, miR-941 expression levels were examined in miRNA profiles of previously sequenced LSCC tissues and in LSCC cell lines, and elevated miR-941 expression was consistent relative to the controls. These findings further indicate that exosomal miRNA expression levels are related to their source cells and can provide pathophysiological insight in lieu of a biopsy 20, 22, 50. These findings collectively further suggest that serum exosomal miR-941 can serve as a potential LSCC biomarker for screening asymptomatic individuals and monitor disease recurrence.

When examining publicly available datasets and the literature 41, miR-941 has been shown to be significantly upregulated in various tumor tissues, including HNSCC and cancer-derived cell lines. These findings indicate that miR-941 most likely plays an important role as a cancer-promoting factor and is associated with tumor pathogenesis. Considering that malignant proliferation and local invasion may be early events in the LSCC progression, the ability of miR-941 to affect proliferation and invasion was examined herein using two LSCC cell lines. As expected, miR-941 promoted LSCC proliferation and invasion. It is generally known that miRNAs play an important role in the regulation of cellular networks in almost all cancers and act as tumor suppressors or oncogenes, subsequently affecting tumorigenesis and the development of various cancers 16, 51, 52.

MiR-941 is a human-specific miRNA that has been reported to have played a crucial role in human evolution and gene regulation involving neurotransmitter signaling, thus influencing human-specific cognitive functions 42. Currently, little is known regarding the role of miR-941 in human cancers. In a study examining hepatocellular carcinoma (HCC), miR-941 was significantly downregulated in tumor tissues, and acted as a tumor suppressor inhibiting cell proliferation, migration and invasion by targeting lysine (K)-specific demethylase 6B (KDM6B) directly 53. However, another group that analyzed the HCC microarray datasets in Gene Expression Omnibus found that miR-941 was upregulated in tumor tissues when compared with normal tissues. Thus, they suggested that the role of miR-941 should be examined further 54. In gastric cancer, miR-941 was also observed to be downregulated, possibly targeting KDM6B and TAO kinase 1 (TAOK1) to inhibit cell proliferation, migration and invasion 55. However, in another recent study, miR-941 expression was examined in 15 healthy human tissues and 8 human cancer cell lines, and miR-941 expression was about 8-fold higher in the tumors 41. Additionally, miR-941 expression increased more than 2-fold when normal melanocytes were transformed into melanomas and miR-941 target genes, mainly tumor suppressor genes, were significantly repressed. In the case of laryngeal cancer, the function of miR-941 has not been reported. Herein, the *in vitro* results showed that miR-941 plays a role in promoting the pathological process of laryngeal cancer, but the downstream mechanism still requires elucidation.

An increasing number of evidence reveals that tumor cells can transfer exosomal miRNAs to surrounding tumor cells through paracrine or autocrine effects, thereby mediating cell-to-cell communication and promoting malignant tumor behavior 56-59. In this study, the expression pattern of miR-941 in FD-LSC-1 and Tu 686 cells was the opposite of their exosomes. We speculate that miR-941 may be encapsulated into exosomes and transferred between tumor cells, thereby promoting laryngeal cancer cell proliferation and invasion as well as promoting a cascade amplification reaction. However, further experimentation is required to clarify these findings.

Our research has some limitations. First, the clinical samples examined were only collected in one center and were not as diverse of a multicenter set. Second, when selecting candidate miRNAs, only upregulated miRNAs with a high abundance were selected, which could have contributed to a loss of other low abundance markers with potential diagnostic value. Additionally, the mechanism governing the role of miR-941 in malignant tumor behavior and the question of whether miR-941 is encapsulated in exosomes to promote laryngeal cancer development still requires further elucidation.

In conclusion, serum exosomal miR-941 shows the potential to be a promising biomarker for LSCC diagnosis and possible targetable factor for LSCC treatment.

## Figures and Tables

**Table 1:** List of Sequences of All Primers in the Present Research

primer name	primer sequence (5'-3')
Hsa-miR-30a-5p-F	GCAGTGTAAACATCCTCGACT
Hsa-miR-532-5p-F	CATGCCTTGAGTGTAGGAC
Hsa-miR-181a-5p-F	CATTCAACGCTGTCGGT
Hsa-miR-425-5p-F	GCAGAATGACACGATCACTC
Hsa-miR-363-3p-F	AGAATTGCACGGTATCCATC
Hsa-miR-424-3p-F	CAAAACGTGAGGCGCT
Hsa-miR-181b-5p-F	GCAGAACATTCATTGCTGTC
Hsa-miR-181a-2-3p-F	GACCACTGACCGTTGAC
Hsa-miR-16-5p-F	CAGTAGCAGCACGTAAATATTG
Hsa-miR-941-F	GCACCCGGCTGTGT
Hsa-miR-27a-5p-F	AGGGCTTAGCTGCTTGT
Hsa-miR-1246-F	CGCAGAATGGATTTTTGGAG
Hsa-miR-452-5p-F	CGCAGAACTGTTTGCAGAG
Hsa-miR-1-3p-F	CGCAGTGGAATGTAAAGAAG
Hsa-miR-7-5p-F	CGCAGTGGAAGACTAGTG
Hsa-miR-3529-3p-F	AACAACAAAATCACTAGTCTTCC
Hsa-miR-24-3p-F	TGGCTCAGTTCAGCAGGAACAG
Hsa-miR-223-5p-F	GCAGCGTGTATTTGACAAG
Universal-R	CTCAACTGGTGTCGTGGA
U6-F	TCGCTTCGGCAGCACATAT
U6-R	ATTTGCGTGTCATCCTTGC

F, forward primer; R, reverse primer.

**Table 2:** MiRNAs Differentially Expressed in LSCC Patients (n = 6) Relative to the HCs (n = 6) Following RNA-seq (*P* < 0.05; | log2 fold-change | ≥ 0.5)

miRNA	LSCC_Ex_readcount	HC_Ex_readcount	log_2_FoldChange	*P*-value
Upregulated miRNA				
hsa-miR-1246	392.891	122.970	1.400	0.000
hsa-miR-27a-5p	299.030	122.568	1.127	0.001
hsa-miR-1291	37.767	10.102	1.398	0.001
hsa-miR-574-5p	61.804	25.432	1.109	0.002
hsa-miR-196a-5p	235.302	48.010	1.443	0.003
hsa-miR-365a-5p	13.963	2.770	1.423	0.003
hsa-miR-7704	18.263	4.184	1.354	0.003
hsa-miR-522-3p	15.708	2.740	1.357	0.006
hsa-miR-767-5p	2.409	0.000	1.302	0.006
hsa-miR-4677-5p	2.954	0.585	1.271	0.010
hsa-miR-452-5p	101.282	55.955	0.768	0.012
hsa-miR-556-5p	12.751	4.314	1.094	0.015
hsa-miR-105-5p	11.412	1.996	1.204	0.015
hsa-miR-548ah-3p	27.558	12.547	0.909	0.017
hsa-miR-548p	27.558	12.745	0.892	0.018
hsa-miR-487b-5p	2.360	0.099	1.122	0.019
hsa-miR-203a-3p	40.489	21.240	0.783	0.020
hsa-miR-203b-5p	40.489	21.240	0.783	0.020
hsa-miR-3128	2.148	0.487	1.099	0.027
hsa-miR-551a	6.673	2.561	1.020	0.027
hsa-miR-1-3p	1564.803	692.038	0.911	0.028
hsa-miR-552-3p	5.304	1.311	1.059	0.033
hsa-miR-4654	3.021	0.446	1.030	0.036
hsa-miR-1185-2-3p	4.428	1.506	1.005	0.039
hsa-miR-2277-5p	4.042	1.080	0.987	0.040
hsa-miR-7-5p	4353.305	2404.923	0.727	0.041
hsa-miR-3529-3p	4290.922	2395.239	0.715	0.043
hsa-miR-941	994.324	478.947	0.822	0.045
hsa-miR-125b-1-3p	23.944	9.010	0.940	0.046
hsa-miR-24-3p	17884.990	12406.933	0.492	0.050
novel_259	2.369	0.198	0.938	0.050
novel_428	2.369	0.198	0.938	0.050
hsa-miR-141-5p	0.966	0.000	0.849	0.050
hsa-miR-223-5p	8001.591	4445.220	0.713	0.050
Downregulated miRNA				
hsa-miR-150-5p	437.928	1442.383	-1.450	0.000
hsa-miR-4685-3p	4.820	17.512	-1.516	0.000
hsa-miR-3173-5p	7.688	19.653	-1.226	0.001
hsa-miR-484	1324.464	2478.547	-0.838	0.001
hsa-miR-204-5p	18.582	53.285	-1.262	0.001
hsa-miR-451a	882525.102	1304912.611	-0.546	0.002
hsa-miR-4745-5p	0.072	2.796	-1.526	0.002
hsa-miR-1306-5p	4.433	14.641	-1.260	0.002
hsa-miR-6815-5p	5.666	15.268	-1.144	0.003
hsa-miR-4732-3p	78.048	182.434	-1.039	0.003
hsa-miR-92a-3p	27155.615	58429.371	-0.962	0.004
hsa-miR-501-3p	697.870	1311.606	-0.821	0.005
hsa-miR-6511b-3p	1.098	5.724	-1.284	0.008
hsa-miR-486-3p	135096.262	240658.136	-0.755	0.009
hsa-miR-483-3p	11.806	32.370	-1.126	0.010
hsa-miR-486-5p	137121.872	241531.605	-0.742	0.010
hsa-miR-6850-5p	0.990	4.660	-1.199	0.014
hsa-miR-6803-3p	0.450	4.295	-1.181	0.017
hsa-miR-6750-5p	2.714	9.667	-1.143	0.017
hsa-miR-1180-3p	481.900	867.740	-0.751	0.019
hsa-miR-550a-3p	1.732	6.826	-1.146	0.021
hsa-miR-550b-2-5p	1.732	6.826	-1.146	0.021
hsa-miR-150-3p	74.077	114.320	-0.584	0.021
hsa-miR-1976	4.705	12.108	-1.008	0.026
hsa-miR-4732-5p	174.145	318.996	-0.755	0.027
hsa-miR-1229-3p	0.424	3.350	-1.087	0.028
hsa-miR-3605-3p	9.607	24.837	-0.981	0.029
hsa-miR-4753-3p	0.436	2.708	-1.060	0.032
hsa-miR-4742-3p	6.878	17.211	-0.920	0.034
hsa-miR-320a	52468.459	97066.421	-0.753	0.035
hsa-miR-592	0.000	1.103	-0.914	0.037
hsa-miR-125a-5p	1558.052	3170.278	-0.819	0.039
hsa-miR-146b-3p	18.873	34.714	-0.758	0.040
hsa-miR-320b	3442.955	6415.482	-0.752	0.040
novel_521	0.177	1.756	-0.964	0.041
hsa-miR-191-5p	11410.077	17378.885	-0.556	0.043
hsa-miR-1228-5p	10.059	20.298	-0.788	0.044
hsa-miR-1285-3p	87.723	144.269	-0.629	0.045
hsa-miR-6833-3p	0.249	1.981	-0.968	0.046
novel_759	0.000	1.378	-0.850	0.048
hsa-miR-3918	0.567	2.484	-0.983	0.048

**Table 3:** Expression Stability Rankings for Candidate Reference Genes Based on BestKeeper, NormFinder, geNorm, the ∆CT and RefFinder Analysis

	BestKeeper			NormFinder		geNorm		∆C_T_ analysis		RefFinder analysis	
Ranking	Gene	SD [±CP]	CV [% CP]	Gene	Stability Value(standard error)	Gene	M value	Gene	Average of STDEV	Gene	Geomean of ranking values
1	U6	0.46	1.74	miR-532-5p	0.509	miR-30a-5p	0.614	miR-532-5p	1.12	miR-30a-5p	1.68
2	miR-30a-5p	0.6	2.63	miR-30a-5p	0.654	miR-181a-5p	0.614	miR-30a-5p	1.17	miR-532-5p	1.86
3	miR-532-5p	0.6	2.03	miR-181a-5p	0.824	U6	0.951	miR-181a-5p	1.25	miR-181a-5p	2.45
4	miR-181a-5p	0.62	2.08	U6	0.831	miR-532-5p	1.012	U6	1.27	U6	2.63
5	miR-425-5p	0.78	2.88	miR-363-3p	0.871	miR-363-3p	1.082	miR-363-3p	1.28	miR-363-3p	5.23
6	miR-363-3p	0.84	3.68	miR-425-5p	1.005	miR-425-5p	1.147	miR-425-5p	1.37	miR-425-5p	5.73
7	miR-424-3p	0.89	2.96	miR-424-3p	1.088	miR-424-3p	1.192	miR-424-3p	1.42	miR-424-3p	7
8	miR-181b-5p	0.98	3.74	miR-181b-5p	1.196	miR-181b-5p	1.247	miR-181b-5p	1.49	miR-181b-5p	8
9	miR-16-5p	1.04	4.69	miR-16-5p	1.396	miR-16-5p	1.335	miR-16-5p	1.64	miR-16-5p	9

SD [±CP], standard deviation of the CP; CV [%CP], coefficient of variance expressed as a percentage of the CP level.

**Table 4:** ROC Curve Analysis of Serum Exosomal miR-941 and miR-27a-5p

miRNAs	Area	SE	*P*-value	95% CI
miR-941	0.797	0.062	<0.001	0.676~0.918
miR-27a-5p	0.672	0.067	0.016	0.540~0.804

**Table 5:** Potential Correlations between Serum Exosomal miR-941 Expression and LSCC Clinicopathological Characteristics

Parameters	Cases (n)	Average Rank	*P*-value^a^
**Age (Years)**			0.954
≤60	24	25.63	
>60	26	25.38	
**Gender**			0.830
Male	46	25.37	
Female	4	27.00	
**Primary Cancer Site**			0.491
Glottic	32	24.00	
Supraglottic	17	27.59	
Subglottic	1	38.00	
**T Staging**			0.731
T1+T2	32	24.97	
T3+T4	18	26.44	
**Cervical Lymph Node Metastasis**			0.108
N0	43	24.16	
N+	7	33.71	
**Clinical Stage**			0.624
I+II	31	24.71	
III+IV	19	26.79	
**Differentiation**			0.164
High	20	20.70	
Medium	18	28.89	
low	12	28.42	

a: Mann-Whitney U test used for two-group analysis. Kruskal-Wallis H test used for three-group analysis.

**Table 6:** MiR-941 Expression in 57 Pairs of LSCC Tissues and Corresponding Normal Tissues Following RNA-seq

miRNA	ACN_readcount	ANM_readcount	log_2_FoldChange	*P*-value
hsa-miR-941	935.950365	451.2506521	1.0388	1.25E-09

ACN, LSCC tissues; ANM, corresponding normal tissues.

## Abbreviations

BLCA: Bladder urothelial carcinoma
BRCA: Breast invasive carcinoma
CESC: Cervical squamous cell carcinoma and endocervical adenocarcinoma
COAD: Colon adenocarcinoma
ESCA: Esophageal carcinoma
HNSC: Head and Neck squamous cell carcinoma
KICH: Kidney chromophobe
KIRC: Kidney renal clear cell carcinoma
KIRP: Kidney renal papillary cell carcinoma
LIHC: Liver hepatocellular carcinoma
LUAD: Lung adenocarcinoma
LUSC: Lung squamous cell carcinoma
PAAD: Pancreatic adenocarcinoma
PRAD: Prostate adenocarcinoma
READ: Rectum adenocarcinoma
SKCM: Skin cutaneous melanoma
STAD: Stomach adenocarcinoma
